# Black phosphorus-doped silk fibroin coating improves osteogenesis for ameliorative graft-bone healing of polyethylene terephthalate artificial ligaments

**DOI:** 10.3389/fbioe.2025.1698237

**Published:** 2026-01-12

**Authors:** Jianxing Wei, Xiulin Wu, Jia Wu, Tianlun Zhang, Jinzhong Zhao, Jiangyu Cai

**Affiliations:** 1 Department of Sports Medicine, Shanghai Sixth People’s Hospital Affiliated to Shanghai Jiao Tong University School of Medicine, Shanghai, China; 2 Medicine & Engineering & Informatics Fusion and Transformation Key Laboratory of Luzhou City, Luzhou, China

**Keywords:** anterior cruciate ligament, artificial ligament, black phosphorus, graft-bone healing, silk fibroin

## Abstract

This study aimed to investigate the effect of black phosphorus-doped silk fibroin (BP/SF) coating on graft-bone healing of artificial ligaments made from polyethylene terephthalate (PET). A BP/SF-coated PET artificial ligament (BP/SF-PET) was prepared, and its surface characteristics were examined using transmission electron microscopy, scanning electron microscopy, Fourier transform infrared spectroscopy, and X-ray diffraction. The cytocompatibility and osteogenic induction ability of the BP/SF-PET ligaments were evaluated *in vitro*. In addition, uncoated PET (PET group, n = 15) and BP/SF-coated PET (BP/SF-PET group, n = 15) artificial ligaments were randomly applied for anterior cruciate ligament reconstruction in rabbits. Micro-computed tomography (CT), and histological, immunofluorescent, and biomechanical analyses were performed 6 and 12 weeks postoperatively to evaluate graft-bone healing *in vivo*. Material characterization results confirmed the presence of BP/SF coating on the PET surface. *In vitro* experiment findings showed that BP/SF coating enhanced the viability of MC3T3-E1 preosteoblasts and L929 fibroblasts, and osteogenic differentiation of MC3T3-E1 preosteoblasts. The BP/SF-PET group exhibited stronger Alizarin Red staining and greater expression of genes involved in osteogenesis (*COL1, OCN*, and *OPN*) than the PET group. Micro-CT and histological analyses showed enhanced graft osseointegration in the BP/SF-PET group, as evidenced by new bone and fibrocartilage tissues developed at the graft-bone interface 12 weeks postoperatively. Accordingly, the histological scores in the BP/SF-PET group were higher. Immunofluorescent staining revealed that positive staining cells for CD68 at 6 weeks were lower and for Wnt-5a and *β*-catenin at 12 weeks were higher in the BP/SF-PET group than those in the PET group. Biomechanical analysis indicated that, at 12 weeks postoperatively, the BP/SF-PET group showed a notably higher failure load and stiffness compared to the PET group. To conclude, BP/SF coating significantly improved the biocompatibility and osteogenesis of PET artificial ligament, facilitating graft-bone integration after anterior cruciate ligament reconstruction via the Wnt/*β*-catenin signaling pathway.

## 1 Introduction

Anterior cruciate ligament (ACL) injuries are among the most prevalent sports-related knee-joint injuries. ACL injury can cause instability of the knee joint, leading to reduced joint movement and functional decline in the early stage and osteoarthritis in the long term (Brophy et al., 2025). ACL injuries cannot self-repair, and arthroscopic ligament reconstruction surgery is currently the most effective method for treating such injuries. Globally, >400,000 ACL injuries require reconstructive surgery annually (Rayan et al., 2015). Nonetheless, in clinical settings, graft selection remains challenging. While autologous grafts are most widely used in clinical practice, issues such as donor site lesions and limited graft sources remain problematic. Allogeneic grafts have defects such as limited donor sources, immune rejection, disease transmission risk, and insufficient mechanical properties (Lin et al., 2020).

Several studies concerning artificial grafts have recently emerged. The Ligament Advanced Reinforcement System (LARS), constructed from non-degradable polyethylene terephthalate (PET), is the predominant artificial ligament used in ligament surgery. However, inadequate hydrophilicity and biocompatibility hinder cell adhesion and tissue integration, often leading to suboptimal graft-bone healing (Tulloch et al., 2019). Several studies have indicated that ACL reconstruction using LARS ligaments may result in complications such as foreign body reactions, bone tunnel enlargement, and graft loosening, affecting its clinical application (Ambrosio et al., 2022; Smolle et al., 2022).

**FIGURE 1:**
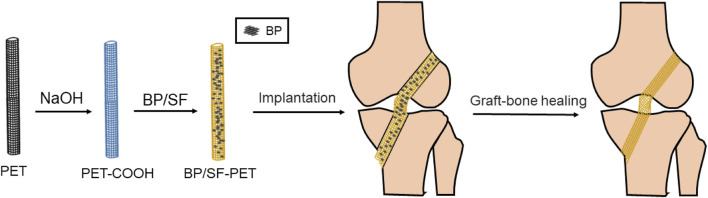
Diagram of the PET artificial ligament with BP/SF coating to improve the graft-bone healing following ACL reconstruction.

Various methods for enhancing the biocompatibility and osteogenic potential of PET artificial ligaments have been proposed, with studies having investigated the modification of bioactive coatings on PET fiber surfaces to enhance the biological performance and improve integration with the host bone (Feng et al., 2025). Previous studies have used diverse bioactive substances, including graphene (Wang et al., 2015), magnesium silicate (Shi et al., 2021), and hydroxyapatite (Chen et al., 2024) for surface modification on PET. However, further studies are required to develop PET ligaments that are coated with homogeneous, stable, and bioactive substances. In recent years, black phosphorus (BP), a novel two-dimensional nanomaterial, has garnered significant attention. Compared with other two-dimensional materials, BP has good biocompatibility and can degrade into nontoxic substances in physiological environments, which are used for the mineralization of bone tissue. BP has also been used in bone tissue engineering (Zeng et al., 2023; Zhang et al., 2023) but not in relation to ligament tissue engineering. This study aimed to explore the effect and underlying mechanisms of BP/SF coating on the osseointegration of these ligaments both *in vivo* and *in vitro*. The impact of BP/silk fibroin (BP/SF) coating on cell viability and differentiation was evaluated *in vitro*, and the *in vivo* study was conducted to investigate the effects of the BP/SF-PET ligament utilizing a rabbit model for ACL reconstruction (Figure 1).

## 2 Methods

### 2.1 Preparation of the BP/SF-PET ligament

To eliminate the sericin, *Bombyx mori* silk was treated in a 0.5 wt% Na_2_CO_3_ solution at 100 °C for 60 min. The degummed silk was washed with distilled water, dried, and dissolved in a CaCl_2_/H_2_O/C_2_H_5_OH solvent at 80 °C for 40 min. At room temperature, dialysis was performed with distilled water for 72 h, followed by filtration and freeze drying to obtain the regenerated SF sponge.

BP crystal powder was sourced from Jiangsu XFNANO Materials Tech Co., Ltd., Nanjing, China. BP nanosheets were produced using a straightforward liquid-phase exfoliation technique. 10 mg of BP crystal powder was dispersed in 10 mL of N-methylpyrrolidone solution (Aladdin Reagents). Afterward, the mixture underwent sonication in an ice bath for 12 h. Subsequently, the resulting brown suspension was centrifuged at 4,000 rpm for 15 min to eliminate the unstripped crystal powder, and the supernatant containing the BP nanosheets was collected. The supernatant was centrifuged at 15,000 rpm for 10 min to remove N-methylpyrrolidone. We then re-dispersed the BP nanosheets in deionized water to prepare a concentration of 100 μg/mL. The SF sponge was then added to the BP dispersion and stirred for mixing, followed by defoaming. The solution was prepared with a concentration of 10 mg/mL.

The PET fabric was obtained from the LARS ligament. The PET fabric was cleaned by immersion in anhydrous ethanol for 30 min, followed by a 10-min ultrasonic treatment at room temperature, washing with deionized water, and drying. The PET fabric was immersed in a 10% NaOH solution, boiled for 60 min, and rinsed thoroughly with deionized water. After drying, the PET fabric was placed in the BP/SF solution, thoroughly stirred, and allowed to react for 24 h. After washing with deionized water and drying, a PET fabric with BP/SF coating was obtained.

### 2.2 Characterization

Transmission electron microscopy (TEM, JEM-2100HR, Japan) at 200 kV was used to analyze the morphology of the BP nanosheets. A TESCAN VEGA3 scanning electron microscope was used to investigate the morphologies of the PET and BP/SF-PET sheets. The samples were coated with gold in a vacuum and observed at an accelerating voltage of 20 kV. A Fourier transform infrared (FTIR) spectrometer (Thermo Fisher Scientific 5225 Verona RD, United States) was used to identify and compare the chemical groups in the samples. Scanning was performed at a resolution of 4 cm^−1^ with the range spanning from 4,000 to 500 cm^−1^. The crystal structure of the samples was analyzed using X-ray diffraction (XRD) with a Rigaku Ultima IV tester with Cu K*α* radiation. The voltage and current were adjusted to 40 kV and 40 A, respectively. The voltage and current were adjusted to 40 kV and 40 A, respectively. The scan covered a range from 5° to 80° (2θ) at a rate of 5° per min.

### 2.3 *In vitro* experiments

#### 2.3.1 Cell viability

Preosteoblast cell lines (MC3T3-E1) and fibroblast cell lines (L929) were kindly provided by Cell Bank, Chinese Academy of Sciences (Shanghai, China). The MC3T3-E1 cells underwent digestion using trypsin and then added to α-MEM medium containing 10% fetal bovine serum and 1% penicillin and streptomycin solution, while the L929 cells were digested with trypsin and then added to Dulbecco’s Modified Eagle’s Medium containing 10% fetal bovine serum and 1% penicillin and streptomycin solution. The MC3T3-E1 and the L929 cells were diluted to a cell suspension of 1 × 10^5^ cells/mL, respectively. PET and BP/SF-PET fabrics were positioned in 24-well plates, with each well receiving 1 mL of MC3T3-E1 or L929 cell suspension, and incubated in a 37 °C constant temperature incubator. After 1, 3, and 5 and 14 days of culture, an assessment of cell viability was performed using a CCK-8 assay. The medium was removed, and the fabrics were rinsed with phosphate buffered saline (PBS). Afterward, medium (360 μL) and CCK-8 solution (40 μL) were introduced to each well and placed in a 37 °C incubator for 4 h. A volume of 100 μL of the supernatant from each well was moved to a 96-well plate, and the absorbance was read at 450 nm with a microplate spectrophotometer (Cai et al., 2025).

#### 2.3.2 Osteogenic differentiation

Osteogenic differentiation was assessed by culturing cells on PET and BP/SF-PET fabrics in an osteogenic induction medium (Cyage, Guangzhou, China).

##### 2.3.2.1 Alizarin Red staining

After culture for 21 and 28 days, the fabrics were rinsed three times with PBS and then fixed in 70% ethanol for 60 min. The samples were washed with PBS, stained for 30 min at room temperature with 0.5% Alizarin Red S (pH 4.2, Sigma-Aldrich, St. Louis, MO, United States), and imaged. Calcium nodule deposition was quantified by treating the cells with 10% cetylpyridine chloride (Sigma-Aldrich, St. Louis, MO) at room temperature for 1 h after 21 and 28 days of culture. A microplate spectrophotometer was used to measure the absorbance of the supernatant at 562 nm.

##### 2.3.2.2 Real-time quantitative polymerase chain reaction

After culturing for 7 and 14 days, TRIzol reagent (Invitrogen, Carlsbad, CA, United States) was used to extract total RNA. RNA was reverse-transcribed into cDNA with a reverse transcription kit. cDNA was synthesized from oligo (dT) primers using a reverse transcriptase kit (Promega). Quantitative polymerase chain reaction (qPCR) was conducted using SYBR Green Premix Ex Taq. The expression levels of the genes, including collagen 1 (*COL1*), osteopontin (*OPN*) and osteocalcin (*OCN*), were adjusted to *β*-actin. The 2^−ΔΔCt^ method was employed for relative quantitative analysis (Tan et al., 2025). Supplementary Table S1 shows the primer sequences.

### 2.4 *In vivo* experiments

#### 2.4.1 A rabbit model for ACL reconstruction

Thirty mature male New Zealand white rabbits (weight, 2.8–3.5 kg) were assigned into two groups of 15 using a computer-generated random number table. ACL reconstruction was performed using uncoated PET ligaments (PET group) or BP/SF-coated PET ligaments (BP/SF-PET group). Anesthesia was induced by an intravenous injection of 3% pentobarbital (1.0 mL/kg). One knee joint was randomly selected as the surgical site. The patella was incised in layers using a medial patellar approach, followed by lateral dislocation to expose and excise the primary anterior ACL. A Kirschner wire with a diameter of 2.5 mm was utilized to create tunnels in the femur and tibia at the original ACL footprint, and the grafts were subsequently inserted into the bone tunnels. The ends of the graft were attached to the adjacent periosteum and soft tissue with sutures. The wound was closed, and the rabbits were allowed to move freely postoperatively. The femur-graft-tibial complex was collected for further examination after the animals were sacrificed by a lethal dose injection of pentobarbital (100 mL/kg for injection) at 6 and 12 weeks postoperatively.

#### 2.4.2 Micro-CT analysis

Twelve weeks postoperatively, the graft-tibial complex was scanned using a Skyscan 1,176 micro-CT imaging system (Bruker, Kontich, Belgium) at a spatial resolution of 35 μm, perpendicular to the long bone axis, with settings of 65 KV and 378 μA, and a 1 mm aluminum filter. The cross-sectional area of the bone tunnel and the bone volume fraction (BV/TV value) were calculated using Data Viewer, CTvol, and CTAn. BV/TV value indicated the fraction of a given volume of interest that is occupied by mineralized bone. The higher BV/TV value represented the better graft-bone healing.

#### 2.4.3 Histological and immunofluorescent evaluation

After being fixed in 10% formalin for 48 h, the samples at 6 and 12 weeks postoperatively were decalcified, dehydrated and then embedded in paraffin. The sample was sliced into 5 μm thick sections perpendicular to the graft’s longitudinal axis using a Leica microtome (Nussloch, Germany). Hematoxylin and eosin (HE), Masson trichrome, and toluidine blue were used to stain the sections of 12 weeks (Qi et al., 2024). Immunofluorescent staining was conducted to assess CD68, Wnt-5a and *β*-catenin expression. Sections were treated with 2% bovine serum albumin at room temperature for blocking. The sections were incubated overnight at 4 °C with anti-CD68, anti-Wnt-5a or anti-*β*-catenin antibodies (both from Abcam), followed by 1 h incubation at 37 °C with corresponding secondary antibodies. After being thoroughly rinsed with PBS, the sections had their nuclei stained with DAPI (Life Technologies, CA, United States). The images were by observed using a fluorescence microscope (IX53; Olympus, Tokyo, Japan), which were then processed with ImageJ software.

#### 2.4.4 Biomechanical testing

An electronic universal testing machine (Instron 5569, Norwood, MA, United States) was applied to test the femur-graft-tibia complex specimens at 6 and 12 weeks postoperatively. All soft tissues with the exception of the ACL graft were meticulously removed. The sample underwent a 1N preload, followed by a loading rate at 10 mm/min until occurrence of graft rupture or extraction from the bone tunnel. The load-deformation curve was utilized to ascertain the failure load and stiffness.

### 2.5 Statistical analysis

Data are expressed as mean ± standard deviation. Statistical analysis was performed using GraphPad Prism software (version 7.0). Data comparisons between two groups were conducted using unpaired *t*-tests. A P-value of less than 0.05 was considered statistically significant.

## 3 Results

### 3.1 Characterization

**FIGURE 2:**
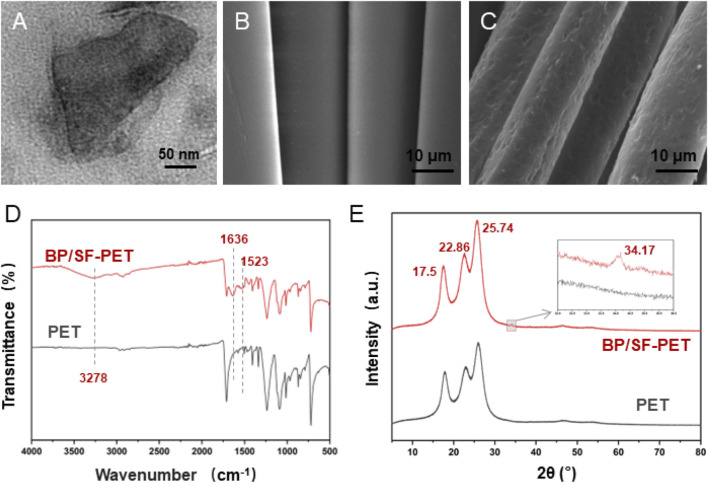
Material characterization. **(A)** TEM images of the morphology of BP, **(B,C)** SEM images of the morphology of PET and BP/SF-PET, **(D)** FTIR spectra of PET and BP/SF-PET, and **(E)** XRD patterns of PET and BP/SF-PET.

The BP nanosheets displayed a consistent two-dimensional sheet structure with an average lateral dimension of 236.3 ± 62.5 nm (Figure 2A). Scanning electron microscopy (SEM) displayed that the surface of the PET fabric was initially flat and smooth (Figure 2B) but became rough and covered by a film layer following BP/SF coating (Figure 2C). The coating thickness was approximately 4–5 μm. FTIR confirmed the presence of SF coating on the PET fibers (Figure 2D). In the BP/SF-PET group, the peak at 3,278 cm^−1^ was linked to hydrogen bonding in the SF. The amide II and amide I peaks for SF were found at 1,523 cm^−1^ and 1,636 cm^−1^, respectively (Srihanam et al., 2010). Figure 2E presents the XRD patterns for both PET and BP/SF-PET. Both groups exhibited typical PET diffraction peaks at 2θ = 17.50, 22.86, and 25.74, as observed in PET and BP/SF-PET samples. Additionally, a typical peak at 2θ = 34.17 was observed for the BP/SF-PET sample, corresponding to the (040) plane of pure BP material (Tiouitchi et al., 2019).

### 3.2 *In vitro* experiments

**FIGURE 3:**
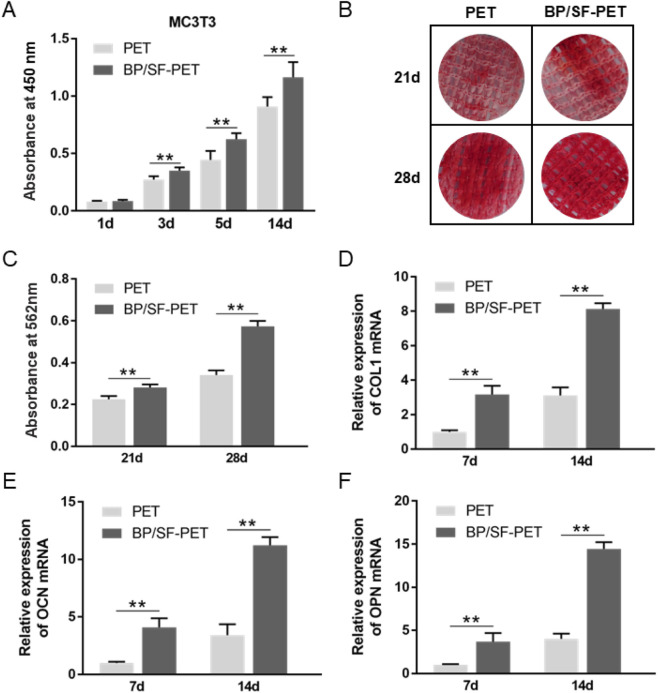
*In vitro* experiments. **(A)** CCK-8 assay of MC3T3-E1 cells cultured for 1, 3, 5 and 14 days in the PET and BP/SF-PET groups; **(B)** ARS staining; **(C)** quantitative analysis of the MC3T3-E1 in the PET and BP/SF-PET groups following 21 and 28 days of osteogenic induction; **(D–F)** RT-qPCR for the relative mRNA expressions of *COL1, OCN*, and *OPN* of MC3T3-E1 cells in the PET and BP/SF-PET groups following 7 and 14 days of osteogenic induction. **P < 0.01.

The CCK-8 assay showed a gradual increase in the MC3T3-E1 and L929 cell number over time in both the PET and BP/SF-PET groups (Figure 3A; Supplementary Figure S1). On culture days 3, 5 and 14 days the BP/SF-PET group exhibited enhanced cell viability, indicating that the BP/SF coating facilitated the proliferation of MC3T3-E1 and L929 cells without cytotoxicity.

Figures 3B,C illustrate that on culture days 21 and 28, the BP/SF-PET group exhibited greater Alizarin Red staining intensity than the PET group. According to quantitative analysis, calcium nodule deposition was significantly higher in the BP/SF-PET group than in the PET group on 21 and 28 days of culture (*P* < 0.01). RT-qPCR analysis indicated that *COL1*, *OCN*, and *OPN* mRNA expression levels were elevated in the BP/SF-PET group compared with those in the PET group on days 7 and 14 (Figures 3D–F). These findings indicate that the BP/SF coating enhanced osteogenic differentiation of MC3T3-E1.

### 3.3 *In vivo* experiments

**FIGURE 4:**
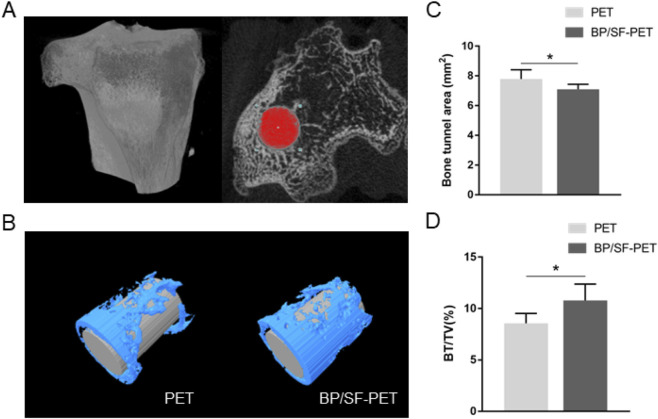
Micro-CT analysis of the PET and BP/SF-PET groups at 12 weeks postoperatively. **(A,B)** The cross-sectional areas of the tibial bone tunnel **(A)** and the reconstructed images **(B)**; **(C,D)** Quantitative analysis of bone tunnel areas **(C)** and BV/TV value **(D)** in the PET and BP/SF-PET groups. *P < 0.05.

Micro-CT analysis revealed that the BP/SF-PET group exhibited a significantly smaller average bone tunnel area (7.1 ± 0.3 mm^2^) compared with the PET group (7.8 ± 0.6 mm^2^, *P* < 0.05), and a significantly higher BV/TV value (10.8% ± 1.6%) than the PET group (8.5% ± 1.0%, *P* < 0.05) (Figure 4).

**FIGURE 5:**
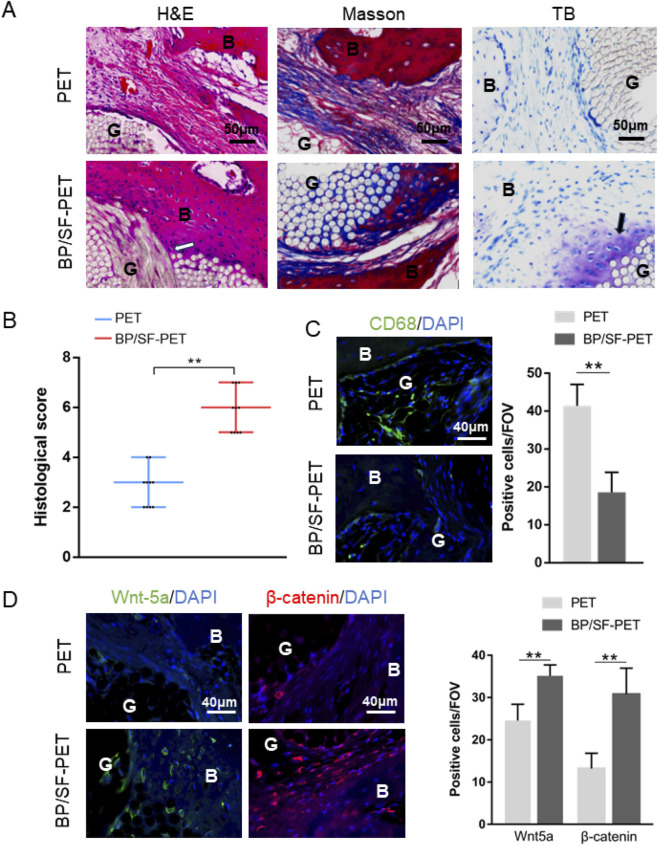
Histological and immunofluorescent results of the PET and BP/SF-PET groups. **(A)** Hematoxylin and eosin, Masson, toluidine blue, and **(B)** histological score of the sections at 12 weeks; **(C,D)** Immunofluorescent staining of **(C)** CD68 at 6 weeks and **(D)** Wnt-5a and *β*-catenin at 12 weeks. The white arrow indicates new bone tissue growth into the graft, and the black arrow indicates fibrocartilage tissue formation at the interface in the BP/SF-PET group. B, bone; G, graft; IF, interface; FOV, field of view.

Histological staining (Figure 5A) showed that, at 12 weeks, lower inflammatory response and new bone tissue in contact and integrated with the graft could be observed in the BP/SF-PET group. More importantly, fibrocartilage tissue formation was observed using toluidine blue staining. Conversely, the PET group exhibited predominantly fibrous scar tissue at the graft-bone interface. Accordingly, the histological score of the BP/SF-PET group (6 ± 1) was higher than that of the PET group (3 ± 1) (*P* < 0.01) (Figure 5B). Immunofluorescent staining revealed that the BP/SF-PET group had less macrophages (CD68^+^ cells) infiltration per field of view compared with the PET group at 6 weeks (19 ± 5 vs. 41 ± 6, P < 0.01) (Figure 5C). Additionally, positive cells per field of view for Wnt-5a and *β*-catenin at the interface were greater in the BP/SF-PET group (35 ± 3, 31 ± 6, respectively) than in the PET group (25 ± 4, 13 ± 4, respectively) at 12 weeks (both *P* < 0.01) (Figure 5D).

**FIGURE 6:**
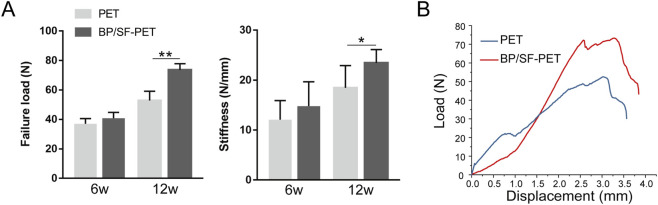
Biomechanical results for the PET and BP/SF-PET groups at 6 and 12 weeks postoperatively. **(A)** The failure load and stiffness in the PET and BP/SF-PET groups, **(B)** Representative load-deformation curves of the PET and BP/SF-PET groups at 12 weeks *P < 0.05; **P < 0.01.

Biomechanical analysis indicated that all samples were extracted from the bone tunnel without any graft fractures. Figure 6 illustrates that, at 6 weeks, the failure load between the PET and BP/SF-PET groups did not differ significantly (37.1 ± 3.5 N vs. 40.8 ± 4.0 N, respectively; *P* = 0.12). At 12 weeks, the BP/SF-PET group exhibited a significantly higher failure load compared with the PET group (73.7 ± 4.2 N vs. 52.9 ± 6.3 N, respectively; *P* < 0.01). At 6 weeks postoperatively, stiffness did not differ significantly between the two groups (14.8 ± 4.9 N/mm vs. 12.1 ± 3.8 N/mm, respectively; *P* = 0.31). After 12 weeks, the BP/SF-PET group exhibited significantly greater stiffness compared with the PET group (23.5 ± 2.7 N/mm vs. 18.5 ± 4.5 N/mm, respectively; *P* = 0.04).

## 4 Discussion

Artificial ligaments can help patients with ACL injuries return to sporting activities as soon as possible, and have promising clinical application prospects. However, currently used PET artificial ligaments have poor biocompatibility and cannot easily induce tissue growth, which requires optimization (Chen et al., 2024). This study focused on the key issue of poor healing between PET artificial ligaments and host bones. Through modification of the BP/SF surface coating, the osteogenic performance of the PET materials was significantly improved, achieving better graft-bone healing.

Surface modification techniques have extensive applications in orthopedics, mainly through physical or chemical methods, for treating material surfaces with substances that have good biocompatibility or bioactivity (Liang et al., 2024). Various coatings, including hydroxyapatite, silk fibroin, gelatin, and bioactive glass, have been used to enhance the host integration of PET materials, thereby improving the performance and longevity of ligament implants (Ai et al., 2017; Cai et al., 2020; Li et al., 2019). Among these, numerous studies have indicated that SF coatings greatly enhance the hydrophilicity and biocompatibility of PET materials. Ai et al. (2017) and Chen et al. (2024) prepared SF coatings on the surface of PET materials and reported that the contact angle of the PET materials after coating was significantly reduced and that hydrophilicity was significantly increased. Cai et al. (2019) and Jiang et al. (2016) reported that PET materials after SF coating could accelerate the adhesion and proliferation of fibroblasts, and that cell compatibility improved significantly. Based on these findings, we doped BP into an SF solution and used a chemical method to apply BP/SF coatings to the surface of PET materials. FTIR and XRD analyses confirmed successful coating of BP/SF on the surface, enhanced material biocompatibility, and also bone tissue ingrowth promotion. The degradation products of BP, phosphates and phosphonates, are important components of the phosphate acid-base pairs in organisms that help maintain stable pH. Previous studies have shown that in phosphonic acid coupling agent modification of HAP nanoparticles, the released phosphate and calcium ions from degradation can provide a favorable environment for cell growth, with no abnormal fluctuations in systemic pH observed throughout the degradation process (Jing et al., 2017). Additionally, research on composite scaffolds containing cyclic phosphates demonstrated that during degradation in phosphate-buffered solution, the system’s pH remained stable between 6.64 and 7.06 over the long term, eventually stabilizing at 7.39 (Shuai et al., 2020). Therefore, the phosphates and phosphonates produced by BP degradation do not cause significant pH changes or related side effects.

*In vitro* studies revealed that the BP/SF coating promoted MC3T3-E1 and L929 cell proliferation on PET materials, thereby improving cytocompatibility. The enhanced cytocompatibility of the BP/SF-PET group can be attributed to the SF coating, which roughened the initially smooth PET fiber surface, thereby facilitating the proliferation and adhesion of the MC3T3-E1 and L929 cells. Furthermore, the components of SF have good biocompatibility and can provide specific sites for cell adhesion (Minoura et al., 1995). The infiltration and adhesion of osteoblasts on PET grafts and their continuous proliferation are crucial for graft osseointegration. BP could additionally promote the proliferation and adhesion of MC3T3-E1 cells (Xu et al., 2021). Introduced BP nanosheets into polyethylene glycol fumarate hydrogels. Cell viability assays along with live/dead and cytoskeletal staining indicated that hydrogels containing specific concentrations of BP nanosheets enhanced MC3T3-E1 cell proliferation and adhesion. Meanwhile, the *in vivo* study also showed that BP/SF-PET group had less macrophages infiltration and displayed lower inflammatory response around the graft at 6 and 12 weeks, indicating BP/SF coating effectively enhanced the biocompatibility of PET.

Furthermore, BP enhanced the osteogenic differentiation of the MC3T3-E1. The degradation of BP nanosheets yields non-toxic phosphate, which contributes to the regulation of the bone tissue microenvironment and facilitates calcium and phosphorus crystal mineralization and deposition, thereby creating a conducive matrix for MC3T3-E1 cell osteogenic differentiation (Qi et al., 2024). Jeon et al. (2021) used a flow self-assembly technique to deposit ultrathin BP coatings on glass substrates and cultured MC3T3-E1 cells. The research verified that the BP coating increased the expression of genes related to osteogenesis (*RUNX2, OCN, OPN,* and Vinculin) in MC3T3-E1 cells, which is consistent with our study findings. Micro-CT and histological analyses indicated that the BP/SF coating significantly promoted new bone formation and integration, confirming its osseointegration efficacy *in vivo*. Additionally, the BP/SF-PET group exhibited higher expression of Wnt5a and *β*-catenin in immunofluorescence staining analysis. A previous study reported that BP quantum dots enhanced the osteogenic differentiation of adipose-derived mesenchymal stem cells through the Wnt/*β*-catenin signaling pathway (He et al., 2024). Similarly, our study findings suggest that the Wnt/*β*-catenin signaling pathway may play a role in BP/SF-induced graft-bone integration.

One striking observation during this present study is that histological analysis showed fibrocartilage formation at the graft-bone interface. Cho et al. (2023) developed an injectable FHE (composed of F127, oxidized hyaluronic acid and poly-ε-L-lysine) hydrogel incorporating BP, which enhanced rat autologous tendon-bone healing, particularly fibrocartilage formation at the tendon-bone interface. Compared with autologous or allogeneic grafts, artificial ligaments face challenges in forming a fibrocartilage transition zone post-implantation (Cai et al., 2018). The healing trends of autologous/allogeneic grafts and artificial ligaments are different as the artificial ligaments were fabricated through chemical and textile technologies. It lacks the natural components of ligaments. In this study, BP facilitated direct bonding between the graft and bone, potentially enhancing chondrogenic differentiation at the interface for fibrocartilage development. Native enthesis is histologically defined as a transitional zone comprising ligaments, fibrocartilage, calcified fibrocartilage, and bone. The fibrocartilage layers function as shock absorbers and reinforce weight-bearing areas (Yambe et al., 2024). Therefore, the multilayer regeneration of bone and fibrocartilage led to superior biomechanical properties of the BP/SF-PET group.

This study has several limitations. First, the stability and degradation rate of the BP/SF coating was not detected in the present study, which warrants further investigation. Second, concerning the animal model, the durations of 6 and 12 weeks were relatively short, thus long-term effects should be examined. Moreover, an SF coating alone group is not included to decouple the effects of SF from the specific contribution of BP. Lastly, since rabbits heal faster than humans, additional research on larger animals is necessary to apply the findings to humans. However, our study provides a promising modification strategy for artificial ligament to facilitate graft-bone healing after ACL reconstruction.

In conclusion, the BP/SF coating effectively enhanced the biocompatibility and osteogenesis of the PET artificial ligaments both *in vivo* and *in vitro* and promoted graft-bone healing through the Wnt/*β*-catenin signaling pathway, thereby improving the biomechanical properties of the grafts after ACL reconstruction.

## Data Availability

The raw data supporting the conclusions of this article will be made available by the authors, without undue reservation.
