# Supplementary material for: Black phosphorus-doped silk fibroin coating improves osteogenesis for ameliorative graft-bone healing of polyethylene terephthalate artificial ligaments
Source: Front Bioeng Biotechnol. 2026 Jan 12;13:1698237. doi: 10.3389/fbioe.2025.1698237 (PMC12832776; doi:10.3389/fbioe.2025.1698237)
Supplement: Supplementary file 1 [file Supplementaryfile1.docx]

**Supplementary Information**

**Black Phosphorus-Doped Silk Fibroin Coating Improves Osteogenesis for Ameliorative Graft-Bone Healing of Polyethylene Terephthalate Artificial Ligaments**

Wei Jianxing^1#^, Wu Xiulin^1#^, Wu Jia^2^, Zhang Tianlun^1^, Zhao Jinzhong^1^*, Cai Jiangyu^1,2^*

1 Department of Sports Medicine, Shanghai Sixth People’s Hospital Affiliated to Shanghai Jiao Tong University School of Medicine, Shanghai, 200233, China;

2 Medicine & Engineering & Informatics Fusion and Transformation Key Laboratory of Luzhou City, Luzhou, 646000, China.)

*Correspondence:

Zhao Jinzhong: Department of Sports Medicine, Shanghai Sixth People’s Hospital Affiliated to Shanghai Jiao Tong University School of Medicine, 600 Yishan Road, Shanghai 200233, China

Email address: jzzhao@sjtu.edu.cn

Cai Jiangyu: Department of Sports Medicine, Shanghai Sixth People’s Hospital Affiliated to Shanghai Jiao Tong University School of Medicine, 600 Yishan Road, Shanghai 200233, China

Email address: caijiangyu1@126.com

#Both authors contributed equally to this work.

**Table S1** Gene-specific primers for COL1, OCN, OPN, and β-actin

| Gene | Primer |
| --- | --- |
| COL1 | 5′-AACAGTCGCTTCACCTACAGC-3′ (forward)  5′-GGTCTTGGTGGTTTTGTGTTCG-3′ (reverse) |
| OCN | 5′-GGACCATCTTTCTGCTCACTCTG-3′ (forward)  5′-TTCACTACCTTATTGCCCTCCTG-3′ (reverse) |
| OPN | 5′-CTTTCACTCCAATCGTCCCTAC-3′ (forward)  5′-CCTTAGACTCACCGCTCTTCAT-3′ (reverse) |
| β-actin | 5′-GAGACCTTCAACACCCCAGC-3′ (forward)  5′-ATGTCACGCACGATTTCCC-3′ (reverse) |


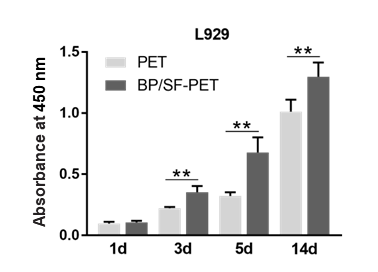


**Figure S1** CCK-8 assay of L929 cells cultured for 1, 3, 5 and 14 days in the PET and BP/SF-PET groups. **P<0.01.
